# Communicating with patients in the age of online portals—challenges and opportunities on the horizon for radiologists

**DOI:** 10.1186/s13244-022-01222-7

**Published:** 2022-05-04

**Authors:** Christoph D. Becker, Elmar Kotter

**Affiliations:** 1 Faculty of Medicine, University of Geneva, Geneva, Switzerland; 2 Department of Diagnostic and Interventional Radiology, Medical Center, University of Freiburg, Freiburg im Breisgau, Germany

**Keywords:** Online patient portals, Professional issues, Patient-centred communication, Patient-centred radiology, Patient-friendly radiology report

## Abstract

The deployment of electronic patient portals increasingly allows patients throughout Europe to consult and share their radiology reports and images securely and timely online. Technical solutions and rules for releasing reports and images on patient portals may differ among institutions, regions and countries, and radiologists should therefore be familiar with the criteria by which reports and images are made available to their patients. Radiologists may also be solicited by patients who wish to discuss complex or critical imaging findings directly with the imaging expert who is responsible for the diagnosis. This emphasises the importance of radiologists’ communication skills as well as appropriate and efficient communication pathways and methods including electronic tools. Radiologists may also have to think about adapting reports as their final product in order to enable both referrers and patients to understand imaging findings. Actionable reports for a medical audience require structured, organ-specific terms and quantitative information, whereas patient-friendly summaries should preferably be based on consumer health language and include explanatory multimedia support or hyperlinks. Owing to the cultural and linguistic diversity in Europe dedicated solutions will require close collaboration between radiologists, patient representatives and software developers; software tools using artificial intelligence and natural language processing could potentially be useful in this context. By engaging actively in the challenges that are associated with increased communication with their patients, radiologists will not only have the opportunity to contribute to patient-centred care, but also to enhance the clinical relevance and the visibility of their profession.

## Key points

- As an increasing number of patients can access their radiology reports and images through electronic patient portals, radiologists and radiology departments should prepare for more direct communication with patients who may have questions about imaging results or request second opinions, especially in the context of advanced imaging studies.
- Patient-centred communication requires simplified, patient-friendly radiology reports using consumer health vocabularies and explanations.
- Patients’ use of online portals creates both challenges and opportunities for radiologists.

## Introduction

The role of the radiologist in the communication of imaging results to patients has been the subject of an ongoing debate over decades [1–6]. More recently, increasing awareness of patients’ rights regarding the ownership of their medical data has sparked a trend towards transparency and patients’ participation regarding their personal diagnostic and therapeutic processes. The slogan “nothing about me without me” has been coined to reflect a key element of patient-centred care [7]. Recent developments in information technology (IT) make it now possible for patients to access all their health data, including radiology reports and images directly online via electronic patient portals. This raises questions regarding patients’ understanding of their imaging results and the role of the radiologist in this context. In the present article, we discuss the potential impact of patient portals on the communication between radiologists and patients.

## Direct communication between patients and radiologists: general aspects

In many European countries, direct contact between the patient and the radiologist is naturally inherent to many imaging procedures, e.g. ultrasonography, clinical mammography, fluoroscopy, paediatric radiology and in vascular and non-vascular interventional radiology. The availability of the medical imaging expert for patient’s questions may also be desirable in the context of certain advanced cross-sectional imaging studies, such as magnetic resonance imaging (MRI) or computed tomography (CT), since it helps to build and maintain trust, to reduce stress and to contribute to patient satisfaction [8–10]. Direct communication has therefore been considered as a part of the radiologists’ service to patients. However, this does not necessarily imply that all radiology results are routinely communicated to the patient by the radiologist [5]. In the traditional model, which is still widely favoured by referring physicians and a majority of patients it is rather the referring physician who communicates the result to the patient [6, 11]. Therefore, many patients of today’s generation may not even be aware of the radiologist’s exact role in the diagnostic process. Despite the obvious advantage of discussing diagnostic and therapeutic aspects of the patient’s problem at the same time and in continuity with the referring physician, there are also drawbacks to this model because it disregards some important facts. Firstly, patients’ specific questions may be beyond the competence of many referrers, especially in the case of advanced imaging studies, e.g. CT and MRI. Secondly, a radiology report cannot be regarded in a similar fashion as a laboratory result because image interpretation is an opinion-based, operator-dependent process which is—at least in part—guided by the history and clinical context provided. Incomplete transmission or omission of relevant facts may lead to suboptimal interpretation of images, emphasising the importance of direct and detailed communication, especially in complex clinical situations. Thirdly, discrepancies between original and secondary readings as well as missed diagnostic findings may occur in radiology just like everywhere else in clinical practice. Active engagement of patients in the diagnostic process and direct communication with the responsible radiologist can not only help to reduce the above-described sources of errors but may also be helpful for patients’ understanding of complex imaging findings. Finally, direct, honest disclosure of discrepancies and corrective measures regarding radiological findings are helpful for establishing and maintaining trust and avoiding confusion [12–15]. This is especially true in complex oncologic settings, where it has been shown that discrepancies between primary and secondary interpretations commonly result in significant actionable differences with regard to treatment [16–18]. Although there appears to be no single best way for communicating imaging results in all clinical settings, there are many good reasons for radiologists to be prepared and make themselves available to discuss their imaging findings with patients if requested to do so [5, 13, 14, 19].

Observations from both Europe and North America indicate that a significant minority of patients may prefer to receive their imaging results or additional information directly from the radiologist [6, 19, 20]. However, several operational barriers exist with regard to radiologists’ direct communication of imaging results to patients. Because of the existing diagnostic workload, many radiology departments cannot allocate sufficient time to radiologists in order to explain imaging findings to their patients nor do they provide dedicated consulting facilities for this purpose. According to a recent survey, over 70% of radiologists from different European countries did not have any specific time nor reimbursement allocated for communicating imaging results to patients. Although most radiologists replied that they enjoyed interaction with patients, the majority preferred not to discuss results with patients because of time constraints and workforce shortage, because of a lack of training in communication or because they believed that the imaging results should be communicated by the referring physician [21]. Since many modern digitised radiology departments are based on picture archiving and communication systems (PACS), the organisation of the radiologists’ workflow may inherently limit or preclude direct patient contact in order to prioritise reporting efficiency. However, this concept reinforces the perception of radiological medical services as a commodity and contributes to a professional image that has been referred to as the “invisible” or “vanishing” radiologist because it undervalues the clinical relevance of imaging expertise and limits the radiologist’s contribution to patient-centred care [14, 22, 23].

## Electronic online portals for health data

Web-based electronic patient portals provide 24-h secure online access to the personal health record. Their goal is to improve transparency and continuity of personal medical information, to reduce the risk of errors and to place the patient in the centre of medical information. Access privileges are granted on a personal basis and secured by username and password and end-to-end encryption in order to guarantee privacy. Access to radiological reports may be considered as a standard feature together with all other text information. However, the possibility to view and download radiological images directly and securely in the original DICOM format is not always possible by using the same patient portal server due to the large data volumes; additional access privileges to the server of the respective local healthcare provider may be necessary for this purpose. Optional features of online portals include the possibility of secure messaging with physicians via communication platforms, to schedule non-urgent appointments, to view safety information and preparatory educational material about procedures or to download consent forms.

Some portals are designed to establish a dialogue with the referring physician and/or the responsible radiologist, either directly via the portal, or by e-mail, or by convening a phone or face-to-face meeting. Predefined electronic messages may be generated in order to alert the patient once a radiological report has become available on the portal. The possibility to define delays for the automatic release of documents may help to adapt the release of information to patients according to different clinical settings. The rules for the release of radiological reports and images are often designed by the institutional leadership rather than by the radiology department providing these documents.

Online portals may be designed for a single or multi-institutional setting. However, exchange of medical data within different European countries must take into consideration that within the framework of the General Data Protection Regulation (GDPR) specific rules with regard to the privacy of medical data may still be defined on a national basis [24].

Owing to differences in healthcare systems throughout Europe, the development and deployment of patient portals by healthcare providers in the different countries are quite variable. National, regional or local solutions may include public or private or both sectors combined [25–27]. Some patient portals have evolved from single institution solutions into multi-institutional networks. For example, experience has been gathered over eight years with a local patient portal emanating from the University Hospital of Geneva (HUG), providing access to local health data [25]. All radiological reports published by the University Hospital Department are accessible and include contact e-mail addresses and phone numbers of the responsible radiologists of the different subspecialties. After an eight-year period including a total of over 50′000 subscribed patients, this local portal has recently been replaced by a regional platform for Western Switzerland including most hospitals and an increasing number of outpatient facilities in the region but also allowing data exchange with other regional networks within the country (www.cara.ch). Certification, patient identification, data protection and funding of this regional network are all based on public law and on non-profit principle. Once subscribed to the service, patients can manage access privileges to their health record themselves. The specific rules for the release of documents are defined by the providing healthcare institutions. All radiological reports are accessible, and selected key images can be included in the form of multimedia documents although direct viewing of complete image files in DICOM format still requires separate access privileges by the individual providing institution. At the Medical Centre of the University of Freiburg a dedicated online portal for radiological images and reports is provided by the radiology department itself. Using the combination of a QR-code and a password patients and referrers physicians can access their images in DICOM format and their reports in DICOM-SR format directly via the PACS server. The University Medical Centre has also established a digital portable application (App: “Meine Uniklinik”) which allows patients of the day clinic of the Interdisciplinary Cancer Centre to receive doctor's letters and appointments via a personalised, secured account. The same application also supports patients and visitors for administrative tasks and provides important timely information, e.g. in the context of the COVID-19 pandemic. Over 200 patients have subscribed so far, and their feedback is used to continuously improve the application and to support new users by building a knowledge base on the corresponding website. An institutional patient portal is to be set up in order to implement this digital approach across the entire University Hospital and at the same time to include physicians in private practice and other external healthcare providers. The future portal aims to implement digital admission, treatment, and discharge management in close digital communication with the existing: “Meine Uniklinik” app, allowing access to radiological images and reports stored in the patient record.

Other institutions in different European countries have deployed dedicated online portals serving similar purposes [26, 27]. As this development continues on larger scale, we may anticipate that patients throughout Europe will eventually be able to access their personal radiological reports and/or images directly online if they wish to do so.

## Impact of online patient portals on the practice of radiology

Although in the past it was not very common for patients to view and discuss their radiology reports or images directly without medical assistance, this may change as future patient generations access their documents increasingly through online portals. Most of the published experience with online access of radiology data has so far been based on patient surveys from North American Institutions, providing some interesting insights regarding the use of radiology reports and images by patients [12, 20, 28–32]. Based on published observations, a large majority of patients prefer the ability to access both radiological reports and images; approximately one-half of the patients who subscribed to patient portal access view their imaging results, with sociodemographic differences being observed in that women and younger patients were most likely to read their radiological reports [28, 33]. The latter observation was confirmed by another study, showing that female patients used online portals for radiological results twice as often as male patients, and that imaging-related inquiries via the portal were significantly more likely to originate from women than from men [31]. It is noteworthy that radiological reports were accessed more frequently via patient portals than clinical notes [33].

In principle, online portals offer the possibility to make radiological reports and images available as soon as they are finalised. Timely communication of imaging results has been identified as an important factor of patient satisfaction. According to some reports, patients may even consider timeliness more important than the question whether it is the radiologist or the referrer who communicates the result of an imaging test [3, 4]. Observations of patient enquiries via the online portal of a large academic medical centre in the USA indicated that most online enquiries related to radiologic imaging were generated in order to obtaining radiology results timely [31]. However, if radiological reports are automatically released on a patient portal upon validation by the radiologist, their online availability often significantly precedes the time by which the referring physician views the report. Delaying direct access of patients to the radiological reports of non-urgent examinations for a few days may allow for sufficient time for the referring physician to study the results before discussing them with the patient. Therefore, institutions may define rules for the automated release of radiology reports, taking clinical settings into consideration in which accompanying medical information usually appears desirable [12, 29]. For example, the results of a skeletal radiography showing a fracture may be released automatically whereas those of a complex oncological follow-up examination may be released only after personal consultation with the referring physician. Some institutions leave the decision to release a radiological report to the referring physician, who must then make it manually available [12, 29, 34]. A recent survey indicated that a delay of three days for release of reports was acceptable for most patients [20]. Nonetheless, the time required by the referring physician to review radiology reports has been identified as a dominant factor for delay in communication according to the traditional model [31].

An important reason for demanding access to radiological reports and images cited by patients is the possibility to share images for second opinions [28]. Portability of health data is a basic requirement of the GDPR which grants patients complete freedom to choose their health care provider [24]. Online portals facilitate the process of obtaining a second opinion about an imaging diagnosis because patients can simply authorise another specialist of their choice to access their images online by using a password.

Access to radiological reports and images by patients without medical assistance raises the question of patients’ understanding of these documents and potential problems in this context. Interestingly, anxiety seems not to be a major concern as it was mentioned only by a small minority of patients when viewing their results and images online without medical explanation [12, 28]. However, incomplete understanding of radiological documents may lead to misinterpretation and confusion in this situation because the medical terms and abbreviations used in current-standard radiological reports may be unfamiliar to many patients, especially in the case of advanced imaging studies such as CT and MRI [20, 29, 30]. In one survey only 27% of patients felt that they clearly understood imaging result when first viewing them by themselves; this percentage rose to 48% when the results were further explained by the referring physician. This was most pronounced in the case of advanced imaging studies since only one of every six patients reported clear understanding of CT or MRI results when first viewing them via the online portal [20]. Unsurprisingly, an analysis of patient enquiries via the online portal also revealed that MRI and CT received most attention [31]. In a patient-centred environment, radiologists can explain imaging results and answer patients’ questions if they can be contacted, either via the online portal or via a professional personal or office e-mail address. According to the experience from academic institutions in the US radiologists’ initial fears of being overwhelmed by interruptions of their workflow by discussions with patients about complex cases appeared unjustified [12].

Another important question addresses the form of the radiological report, which is today still considered by many radiologists as a prosaic piece or art with subjective individual wording. As the use of online patient portals increases, the modern radiologist will have two different audiences for his reporting, namely medical colleagues and patients. Regarding the medical audience, structured, quantitative, actionable reporting has been identified as a key element to increase the clarity, precision and comparison of radiological reports, to enhance multidisciplinary collaboration and to facilitate coding and data mining for clinical research [11, 35, 36]. Therefore, it appears important to introduce disease-specific automated templates for structured reporting where they are particularly useful, e.g. in oncological imaging including internationally standardised criteria for follow-up such as the response evaluation criteria for solid tumours (RECIST) [37]. Once successfully adopted, structured reporting may greatly reduce ambiguity as opposed to traditional narratives. Structured reporting may also be seen as an important first step paving the way towards integrated reporting, which aims to confront and combine the radiological imaging information with other diagnostic methods used in personalised medicine, i.e. digitised pathology or genomics. On the other hand, since the information used in structured reporting templates addresses a medical audience, it is not primarily designed to improve patients’ understanding of radiologic reports. Patient-centred communication rather requires simplified texts using consumer health vocabularies [12, 38–41]. Creating two different formal reports in clinical routine appears unrealistic for radiologists in most European institutions because of the associated workload. Software tools based on artificial intelligence and using natural language processing could potentially be helpful to improve patients’ understanding of electronic health records in the future. For example, dedicated text processing solutions could help to explain medical terms in simplified language or dedicated structured text elements could be used for explaining common diagnostic findings in consumer health vocabulary. Some developments have been reported that are aiming at providing an automated transformation of complete radiological reports into a consumer health vocabulary in English language [40–43]. Hyperlinks could also be included in reports, leading to explanatory websites and multimedia support related to anatomy, physiology, pathology or radiologic examination technique. However, software tools for the different above-mentioned purposes have not yet gained widespread use in radiologic services in Europe and their implementation in daily practice is likely to require considerable efforts in terms of customisation regarding different national and local cultures and languages.

## Challenges and opportunities

As the deployment of online portals for personal health data is likely to increase in Europe in the near future, challenges and opportunities may arise for both patients and radiologists. Even if the majority of patients preferred to adhere to the traditional model of referrer-based information, radiologists should be prepared to answer the questions of the significant minority of patients who prefers direct explanations of imaging results or a second opinion about their radiology results viewed online. Although radiologists are not always involved in establishing the rules and delays for the online release of medical documents on an institutional patient portal, they should at least be aware of the rules of their institution with regard to when and how their reports and images are released for patients’ access. Radiology departments may also have to review their medical workflow and organisation in order to define the most efficient, secure and cost-effective way of communication between patients and radiologists [32]. Although electronic communication tools probably offer the most practical solutions for discussing imaging results with patients, phone conversations or face-to-face meetings may be appropriate in some situations, e.g. complex oncologic settings. In the same context, radiologists’ communication skills are likely to gain importance in the future. Although the vast majority of radiologists responding to the European survey identified the importance of specific training how to communicate significant imaging findings to patients, and although the corresponding learning objectives are described in the European Training Curriculum, only one-fourth of respondents of the survey had received formal radiology-specific training in communication with patients [21, 44].

As patients become a new audience for radiological reports, the traditional narrative description of findings as we know it today may therefore no longer be sufficient as the radiologist's main final product. Radiology reports destined to referrers and other medical colleagues require increasingly specific, structured, actionable and quantitative information. In contrast, patient-friendly reports are rather summaries of imaging findings in consumer health language, ideally including multimedia support and explanatory hyperlinks in order to understand their radiological reports and the images on which they are based. However, the technical and operational hurdles involved in the process of developing and integrating software solutions for this purpose into daily routine should not be underestimated. Last but not least, the linguistic and cultural diversity in Europe demands for country-specific collaboration between patient representatives, radiologists and software developers.

## Summary

As future generations of patients become more involved in decision-making regarding their healthcare, they may also increasingly consult radiology reports and images through patient portals. A significant and perhaps increasing subset of patients may prefer to discuss complex imaging findings with the radiologist who is responsible for the diagnosis. By engaging actively in the challenges that are associated with increased communication with their patients, radiologists will not only have the opportunity to contribute to patient-centred care, but also to enhance the clinical relevance and the visibility of their profession.

## Data Availability

No original data have been used.

## Abbreviations

COVID-19: Coronavirus disease of 2019
CT: Computed tomography
DICOM: Digital imaging communication format
GDPR: General Data Protection Regulation
HUG: Hôpitaux Universitaires Genève
IT: Information technology
MRI: Magnetic resonance imaging
PACS: Picture archiving and communication system
RECIST: Response evaluation criteria in solid tumours

## References

[1] Levitsky DB, Frank MS, Richardson ML (1993). How should radiologists reply when patients ask about their diagnoses? A survey of radiologists' and clinicians' preferences. AJR Am J Roentgenol.

[2] Safdar N, Shet N, Bulas D (2011). Handoffs between radiologists and patients: threat or opportunity?. J Am Coll Radiol.

[3] Basu PA, Ruiz-Wibbelsmann JA, Spielman SB (2011). Creating a patient-centered imaging service: determining what patients want. AJR Am J Roentgenol.

[4] Pahade J, Couto C, Davis RB (2012). Reviewing imaging examination results with a radiologist immediately after study completion: patient preferences and assessment of feasibility in an academic department. AJR Am J Roentgenol.

[5] Berlin L (2009). Communicating results of all outpatient radiologic examinations directly to patients: the time has come. AJR Am J Roentgenol.

[6] Cabarrus M, Naeger DM, Rybkin A (2015). Patients prefer results from the ordering provider and access to their radiology reports. J Am Coll Radiol.

[7] Barry MJ, Edgman-Levitan S (2012). Shared decision making–pinnacle of patient-centered care. N Engl J Med.

[8] Bazzocchi M (2012). Doctor-patient communication in radiology: a great opportunity for future radiology. Radiol Med.

[9] Gutzeit A, Heiland R, Sudarski S (2019). Direct communication between radiologists and patients following imaging examinations. Should radiologists rethink their patient care?. Eur Radiol.

[10] Margulis AR, Sostman HD (2004). Radiologist-patient contact during the performance of cross-sectional examinations. J Am Coll Radiol.

[11] Bosmans JML, Peremans L, Menni M, De Schepper AM, Duyck PO, Parizel PM (2011). How do referring clinicians want radiologists to report? Suggestions form the COVER survey. Insights Imaging.

[12] Lee CI, Langlotz CP, Elmore JG (2016). Implications of direct patient online access to radiology reports through patient web portals. J Am Coll Radiol.

[13] Cox J, Graham Y (2020). Radiology and patient communication: if not now, then when?. Eur Radiol.

[14] Itri JN (2015). Patient-centered radiology. Radiographics.

[15] Maskell G (2019). Error in radiology-where are we now?. Br J Radiol.

[16] Lakhman Y, D'Anastasi M, Micco M (2016). Second-opinion interpretations of gynecologic oncologic MRI examinations by sub-specialized radiologists influence patient care. Eur Radiol.

[17] Spivey TL, Carlson KA, Janssen I (2015). Breast imaging second opinions impact surgical management. Ann Surg Oncol.

[18] Lysack JT, Hoy M, Hudon ME (2013). Impact of neuroradiologist second opinion on staging and management of head and neck cancer. J Otolaryngol Head Neck Surg.

[19] Capaccio E, Podesta A, Morcaldi D (2010). How often do patients ask for the results of their radiological studies?. Insights Imaging.

[20] Garry K, Blecker S, Saag H (2020). Patient experience with notification of radiology results: a comparison of direct communication and patient portal use. J Am Coll Radiol.

[21] European Society of Radiology (ESR) (2020). The identity and role of the radiologist in 2020: a survey among ESR full radiologist members. Insights Imaging.

[22] Glazer GM, Ruiz-Wibbelsmann JA (2011). The invisible radiologist. Radiology.

[23] Brady AP (2021). The vanishing radiologist-an unseen danger, and a danger of being unseen. Eur Radiol.

[24] European Society of Radiology (2017). The new EU general data protection regulation: what the radiologist should know. Insights Imaging.

[25] Rosemberg A, Schmid A, Plaut O (2016). MonDossierMedical.ch—the personal health record for every Geneva citizen. Stud Health Technol Inform.

[26] Rahbek Norgaard J (2013). E-record—access to all Danish public health records. Stud Health Technol Inform.

[27] Otte-Trojel T, de Bont A, Aspria M (2015). Developing patient portals in a fragmented healthcare system. Int J Med Inform.

[28] Halaska C, Sachs P, Sanfilippo K (2019). Patient attitudes about viewing their radiology images online: preintervention survey. J Med Internet Res.

[29] Gefen R, Bruno MA, Abujudeh HH (2017). Online portals: gateway to patient-centered radiology. AJR Am J Roentgenol.

[30] Alarifi M, Patrick T, Jabour A (2020). Full radiology report through patient web portal: a literature review. Int J Environ Res Public Health.

[31] Mervak BM, Davenport MS, Flynt KA (2016). What the patient wants: an analysis of radiology-related inquiries from a web-based patient portal. J Am Coll Radiol.

[32] Liao GJ, Lee CI (2018). Viewing the value of radiology through patient web portals. Acad Radiol.

[33] Miles RC, Hippe DS, Elmore JG (2016). Patient access to online radiology reports: frequency and sociodemographic characteristics associated with use. Acad Radiol.

[34] Okawa G, Ching K, Qian H (2017). Automatic release of radiology reports via an online patient portal. J Am Coll Radiol.

[35] Ganeshan D, Duong PT, Probyn L (2018). Structured reporting in radiology. Acad Radiol.

[36] European Society of Radiology (2018). ESR paper on structured reporting in radiology. Insights Imaging.

[37] Spînu-Popa EV, Cioni D, Neri E (2021). Radiology reporting in oncology—oncologists’ perspective. Cancer Imaging.

[38] Johnson AJ, Easterling D, Williams LS (2009). Insight from patients for radiologists: improving our reporting systems. J Am Coll Radiol.

[39] Martin-Carreras T, Cook TS, Kahn CE (2019). Readability of radiology reports: implications for patient-centered care. Clin Imaging.

[40] Alarifi M, Patrick T, Jabour A (2021). Designing a consumer-friendly radiology report using a patient-centered approach. J Digit Imaging.

[41] Slanetz PJ, Krishnaraj A, Lee CI (2019). Patient portals and radiology: overcoming hurdles. J Am Coll Radiol.

[42] Polepalli Ramesh B, Houston T, Brandt C (2013). Improving patients' electronic health record comprehension with NoteAid. Stud Health Technol Inform.

[43] Qenam B, Kim TY, Carroll MJ (2017). Text simplification using consumer health vocabulary to generate patient-centered radiology reporting: translation and evaluation. J Med Internet Res.

[44] ESR training curriculum for radiology level I 2021. http://www.myesr.org

